# 

**Published:** 1802-11-01

**Authors:** 


### To CORRESPONDENTS.

*Communications are received from Messrs. Pears, Rigg, Burrows, Westall, Philo-Medicus, Indagator, Alpha, Philadelphus, &c.*

*We are sorry to be under the necessity of reminding our Correspondents, that Communications which are intended to be published under assumed Names, must always be accompanied with a private notice to the Editors of the real name and address of the Writer.*

